# SERS detection of surface-adsorbent toxic substances of microplastics based on gold nanoparticles and surface acoustic waves

**DOI:** 10.1039/d3ra07382c

**Published:** 2024-01-09

**Authors:** Hyeong Min Ahn, Jin Oh Park, Hak-Jun Lee, Cheonkyu Lee, Honggu Chun, Kwang Bok Kim

**Affiliations:** a Digital Health Care R&D Department, Korea Institute of Industrial Technology (KITECH) 89, Yangdaegiro-gil, Ipjang-myeon, Seobuk-gu Cheonan 31056 Republic of Korea kb815kim@kitech.re.kr; b Smart Manufacturing System R&D Department, Korea Institute of Industrial Technology (KITECH) 89, Yangdaegiro-gil, Ipjang-myeon, Seobuk-gu Cheonan 31056 Republic of Korea; c Carbon Neutral Technology R&D Department, Korea Institute of Industrial Technology (KITECH) 89, Yangdaegiro-gil, Ipjang-myeon, Seobuk-gu Cheonan 31056 Republic of Korea; d Department of Biomedical Engineering, Korea University 145, Anam-ro, Seongbuk-gu Seoul 02841 Republic of Korea chunhonggu@korea.ac.kr

## Abstract

Microplastics adsorb toxic substances and act as a transport medium. When microplastics adsorbed with toxic substances accumulate in the body, the microplastics and the adsorbed toxic substances can cause serious diseases, such as cancer. This work aimed to develop a surface-enhanced Raman spectroscopy (SERS) detection method for surface-adsorbent toxic substances by forming gold nanogaps on microplastics using surface acoustic waves (SAWs). Polystyrene microparticles (PSMPs; 1 μm) and polycyclic aromatic hydrocarbons (PAHs), including pyrene, anthracene, and fluorene, were selected as microplastics and toxic substances, respectively. Gold nanoparticles (AuNPs; 50 nm) were used as a SERS agent. The Raman characteristic peaks of the PAHs adsorbed on the surface of PSMPs were detected, and the SERS intensity and logarithm of the concentrations of pyrene, anthracene, and fluorene showed a linear relationship (*R*^*2*^ = 0.98), and the limits of detection were 95, 168, and 195 nM, respectively. Each PAH was detected on the surface of PSMPs, which were adsorbed with toxic substances in a mixture of three PAHs, indicating that the technique can be used to elucidate mixtures of toxic substances. The proposed SERS detection method based on SAWs could sense toxic substances that were surface-adsorbed on microplastics and can be utilized to monitor or track pollutants in aquatic environments.

## Abstract 2

SAW-aggregated gold nanoparticles as SERS substrates for detecting surface-adsorbent toxic substances on microplastics.

## Graphical abstract

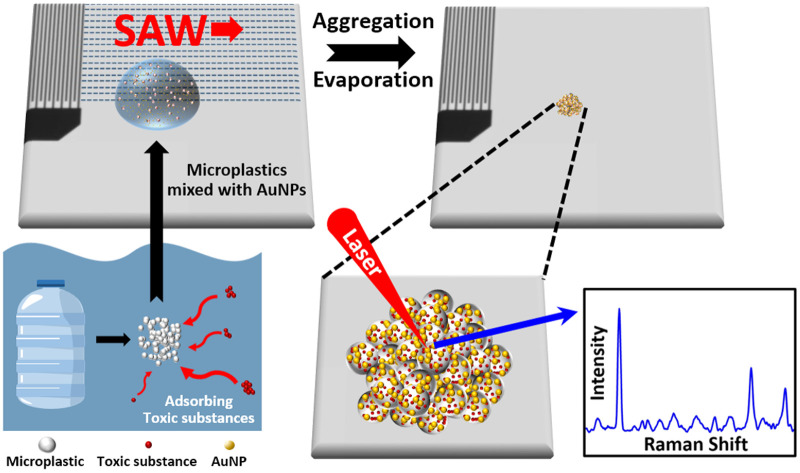

## Introduction

Microplastics have become a growing environmental concern due to their ubiquitous presence in ecosystems^1^ and potential to adsorb toxic substances such as polycyclic aromatic hydrocarbons (PAHs) and persistent organic pollutants (POPs).^2^ These toxic substances pose significant risks to aquatic life, terrestrial organisms, and human health;^3^ for instance, they can bioaccumulate in organisms through the food chain, resulting in adverse effects on reproduction and immune functions.^4^ Furthermore, the widespread distribution of microplastics carrying toxic substances can contaminate natural resources, including water and soil, impacting agriculture and food safety.^5^ Traditional methods to detect toxic substances adsorbed on microplastics include gas chromatography-mass spectrometry (GC-MS),^6^ liquid chromatography-mass spectrometry (LC-MS),^7^ and inductively coupled plasma mass spectrometry (ICP-MS),^8^ which involve extracting and separating the adsorbed substances from the microplastic particles, and then detecting and quantifying them with these techniques.^9^ Although these methods are widely used, they have some limitations. First, the sample preparation process can be time-consuming and labor-intensive.^10^ Extraction and separation procedures require the use of solvents and other chemicals, which may introduce contamination or errors.^11^ Second, the sample preparation process often involves dissolving or destroying the microplastic particles, making it difficult to study the microplastics themselves and their interactions with the adsorbed substances.^12^ More recently, alternative techniques have been proposed for detecting toxic substances, including the capillary zone electrophoresis method,^13^ and optical and spectroscopic methods such as surface-enhanced Raman spectroscopy (SERS).^14,15^

Relying on metallic nanostructures, SERS is a highly sensitive technique that enhances the Raman scattering of molecules.^16^ The SERS signal diminishes exponentially with increasing distance from the metallic surface. Surface plasmon resonance triggers intense Raman scattering from a specific target species within an ideal spatial region of 0–4 nm, where the electromagnetic fields are localized.^17^ SERS uses surface plasmonic resonance to detect molecular fingerprints, even at low concentrations,^18^ and has found applications across multiple domains, including bioengineering, materials science, food science, and environmental monitoring.^19–22^ Nevertheless, SERS faces challenges such as the intricate and laborious process of forming nanostructures,^21^ inconsistent reproducibility resulting from random hotspot generation,^23^ and the demand for specialized knowledge and lengthy procedures to create functional groups on metallic surfaces.^24^ Hence, there is a need to simplify the detection process and nanostructure development.

Traveling waves that move along the surface of piezoelectric substrates, surface acoustic waves (SAWs), have traditionally been used in electronics and mechanics for purposes such as sensors.^25,26^ Rayleigh waves, a specific mode of surface traveling waves, are employed in microfluidics as they can efficiently transfer energy to fluids with minimal loss.^27^ Significant research has been conducted on using SAW-based microfluidics to manipulate and modulate particles.^28,29^ Acoustofluidics, an emerging field, is characterized by small sample requirements, rapid processing, sensitivity, and selectivity.^30^ As SAW has gained attention in the field of bio-applications, studies have been conducted on combining it with other technologies.^31^

**Fig. 1:**
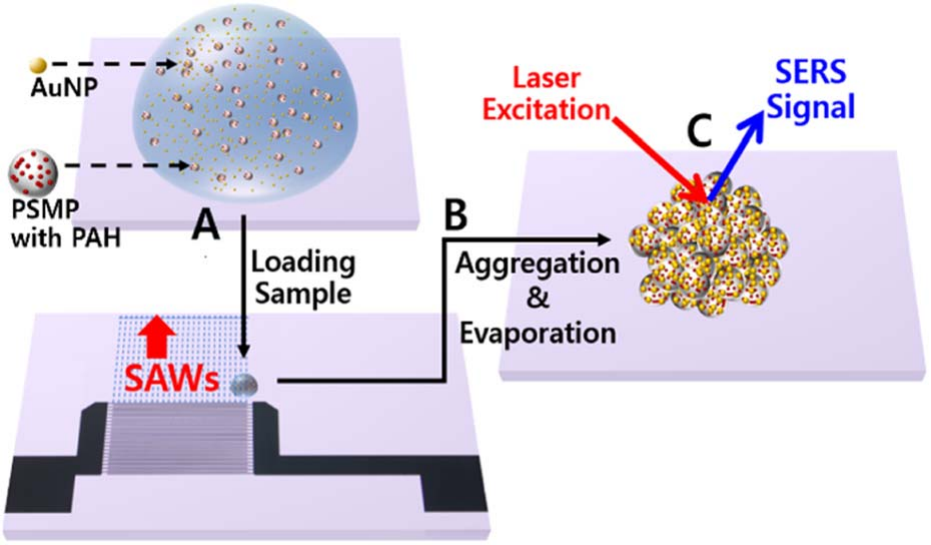
Schematic representation of detecting polycyclic aromatic hydrocarbons (PAHs) adsorbed on polystyrene microparticles (PSMPs) using surfaced-enhanced Raman spectroscopy (SERS) based on surface acoustic waves (SAWs). (A) A 1 μL droplet consisting of 1 μm PSMPs and 50 nm gold nanoparticles (AuNPs) is loaded on the SAW chip. (B) After being aggregated and evaporated completely by the SAWs, (C) SERS signals are measured from the cluster with 785 nm laser excitation on 1 mW for 1 s.

In this work, we propose a new approach to improve the detection of toxic substances adsorbed on microplastic surfaces using SERS combined with SAW technology (Fig. 1). We aimed to form gold nanogaps on microplastic surfaces by aggregating particles based on SAWs to enhance Raman scattering.

This proposed method does not require functionalized gold nanoparticles and a process of extracting toxic substances from microplastic surfaces.^12,24^ In addition, since SAWs are adopted instead of inorganic salts to induce particle aggregation,^29,32^ it is possible to prevent problems that the coatings and types of microplastics have a limited effect on particle aggregation depending on the concentration of inorganic salts.^33–36^

Experiments were conducted by changing variables, such as hydrophobic coating and SAW application, to determine suitable conditions for particle aggregation.

## Experimental

### Methodology

A droplet containing 1 μm polystyrene microparticles (PSMPs) adsorbed with PAHs and 50 nm gold nanoparticles (AuNPs) was loaded onto the SAW substrate without any extraction processes (Fig. 1A). When SAWs were transmitted into the droplet, the particles experienced rotation and aggregation at the center of the droplet due to the internal acoustic streaming induced by SAWs (Fig. 1B). After the droplet evaporated completely, SERS signals were measured from a cluster of PSMPs and AuNPs (Fig. 1C). Characteristic peaks of PAHs adsorbed on PSMPs were identified in the collected SERS spectra.

### Chemicals and materials

In order for SAWs to propagate, piezoelectric substrates are necessary on which the SAWs can proceed. These piezoelectric substrates have different physical properties based on the direction of the material and generate a piezoelectric phenomenon in which dielectric polarization occurs by mechanical force.^27^ Rayleigh waves are a type of acoustic wave mode that is longitudinal and has minimal energy loss during transfer. When these waves encounter a solid–liquid interface, they cause energy attenuation.^37^ This property makes Rayleigh waves ideal for transferring attenuated energy from a solid to a liquid, which is why they are commonly used in microfluidic applications. To generate high-efficiency Rayleigh waves, a piezoelectric substrate made of LiNbO_3_ is often preferred because of its minimal energy loss, manageable handling, and adequate electromechanical interaction in the MHz wavelength range.^28,29^

A 4-inch lithium niobate wafer (LiNbO_3_, 128° Y-cut X-propagating, 500 μm thickness, SAW grade, 2 sides polished; MTIKorea, Republic of Korea) was used as a piezoelectric material for SAWs. PAHs, including pyrene (C_16_H_10_, 98%), anthracene (C_14_H_10_, 99%), and fluorene (C_13_H_10_, 98%), were used as toxic substances. PSMPs (1 μm, 10 wt%, aqueous suspension) were used as microplastics. AuNPs (50 nm, 3.5 × 10^10^ particles per mL, stabilized suspension in citrate buffer) were used as a SERS agent. All materials were purchased from Sigma-Aldrich (St. Louis, Missouri, USA). Other chemicals used in this work include acetone (C_3_H_6_O, 99.8%; DAEJUNG, Republic of Korea), ethanol (C_2_H_6_O, 99.9%; DUKSAN, Republic of Korea), methanol (CH_4_O, 99.8%; DUKSAN, Republic of Korea), AZ 1512 photoresist, and AZ 300 MIF developer (EMD Performance Materials Corp., Germany), and trichloro (1H,1H,2H,2H-perfluorooctyl)silane (C_8_H_4_Cl_3_F_13_Si, ≥97%; Sigma-Aldrich, USA). Ultrapure water (18.3 MΩ cm; HumanScience, Republic of Korea) was used throughout the experiments.

### Preparation of samples

Stock solutions of PAHs were prepared by dissolving them in methanol at an initial concentration of 10 mM and diluting them to the desired concentration with methanol. PSMPs immersed in PAH solution were shaken at 150 rpm for 72 h to adsorb PAH.^38,39^ The PSMPs adsorbed with PAH were separated by centrifugation and rinsed with ultrapure water several times. The concentration of PSMP aqueous suspension was 1.8 × 10^8^ particles per mL. The AuNP suspension was redispersed in ultrapure water to prevent Raman scattering due to citrate buffer and the concentration of AuNP aqueous suspension was 2.1 × 10^11^ particles per mL. The concentration of each suspension was optimized so that AuNPs would be sufficiently distributed on the surface of PSMPs. The PSMP and AuNP aqueous suspensions were mixed for experiments. All processes were conducted at room temperature.

### Fabrication of SAW chips

**Fig. 2:**
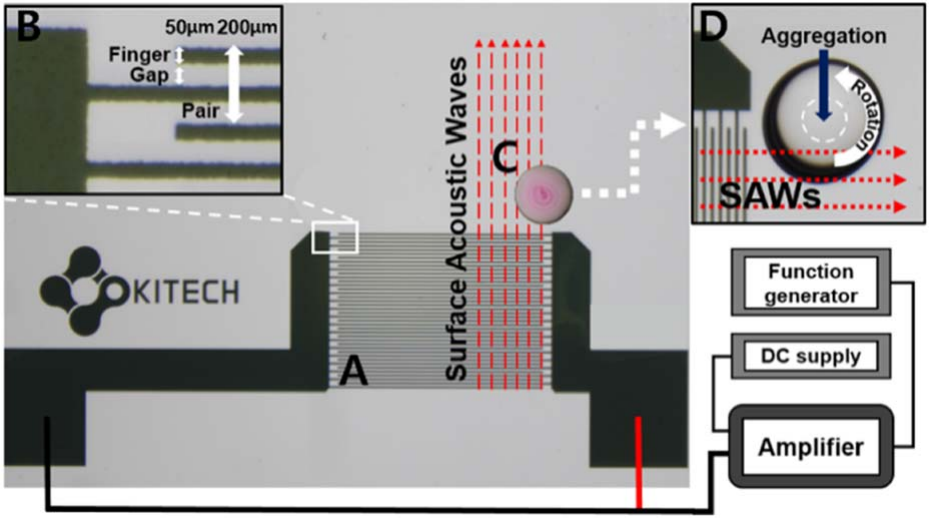
Schematically illustration of the particle aggregation on the SAW chip. (A) Interdigital transducers (IDTs) consist of 20 pairs of 10/50 nm Cr/Au electrode arrays. (B) The width of a finger and the gap between fingers is 50 μm. And the width of a pair consisting of two fingers and two gaps is 200 μm. 19.04 MHz and 19.4 V are applied to the IDTs to generate SAWs. (C) The SAWs propagate along the LiNbO_3_ surface and half of the droplet should be located in the SAW propagation pathway. (D) The droplet revolves and particles in the droplet are aggregated due to internal flow induced by the SAWs.

A 10 nm thick layer of chromium and a 50 nm thick layer of gold were deposited on a LiNbO_3_ substrate by standard UV photolithography with a sputter (ALPS-C03; Alpha-Plus, Republic of Korea) and lift-off processes using AZ 1512 photoresist and AZ 300 MIF developer.^40^Fig. 2A shows the deposited Cr/Au layer, consisting of 20 pairs of interdigitated electrode arrays, acts as an interdigital transducer (IDT). The width of a finger and the gap between fingers is 50 μm. The width of the pair, consisting of two fingers and two gaps, is 200 μm, as determined by the operating wavelength and chip size (Fig. 2B). The shorter the operating wavelength, the shorter the width of the IDT finger and gap, making it possible to miniaturize the chips, but with difficulty in accurately depositing the IDTs.^28^ The LiNbO_3_ wafer was diced into 16 chips of size 2.07 cm × 1.67 cm using a dicing saw (DAD3221; DISCO, Japan). A hydrophobic coating was applied to the diced chips to aggregate the particles efficiently. This improves SERS sensitivity by overcoming the diffusion of analytes in aqueous solutions.^41^ The surfaces of the diced chips were etched using O_2_ plasma (CUTE; FEMTO SCIENCE, Republic of Korea) for 10 min at 100 W. This helps to form surface hydroxyl groups on LiNbO_3_ for chemical reactions with trichlorosilane molecules.^42^ Three chips were placed in a vacuum chamber along with a quartz glass dish containing 60 μL of trichlorosilane solution. They were kept under vacuum (20 kPa) at room temperature for >6 h.^43^ The chips were then rinsed with ethanol and ultrapure water and then dried with nitrogen.

### Characterization

An optical microscope (SMZ1270; Nikon, Japan) was used to acquire images of the evaporation and aggregation processes. The morphologies of the PSMPs and AuNPs were analyzed using scanning electron microscopy (SEM; JSM-6701F; JEOL, Japan). To generate SAWs, a function generator (AFG1062; Tektronix, USA), DC supply (ODP3032; OWON, China), and amplifier (ZHL-1-2W+; Mini-Circuits, USA) were used. Raman spectra were recorded using confocal Raman spectroscopy (FEX; NOST co., Ltd, Republic of Korea) with an excitation wavelength of 785 nm, resolution of 2.5 cm^−1^, beam diameter of 3 μm, and exposure time of 1 s. The laser power applied to the sample was 1 mW. RAON-Spec software (NOST Co., Ltd, Republic of Korea) was used to acquire all SERS spectra. Baseline removal and smoothing of all the data were processed using RAON-Vu (NOST Co., Ltd, Republic of Korea). A mean difference equation with a window size of 5 and a Savizky-Goray filter with a window size of 9 was utilized.

### Propagation of SAWs for particle aggregation

The device setup to generate SAWs and the particle aggregation process by SAW propagation are shown in Fig. 2. The voltage from the DC supply, applied to the IDTs related to SAW energy, was 19.4 V. And the applied sinusoidal signal from the function generator was 19.04 MHz, determined by the acoustic streaming velocity of piezoelectric material divided by the operation wavelength of IDTs.^27^ In this case, the acoustic streaming velocity of LiNbO_3_ is ∼3800 m s^−1^, and the operation wavelength of the IDTs is 200 μm. Fig. 2C shows that the SAWs generated from the IDTs are propagated along the LiNbO_3_ surface until they reach a droplet, in which case the SAWs are reflected and refracted at the interface between LiNbO_3_ and the droplet. In this process, some of the energy of the SAWs is transferred to the droplet and converted into kinetic energy, which causes fluctuations in the droplet, and the droplet revolves along the direction of SAW propagation (Fig. 2D).^29^ The position of the droplet in the SAW propagation pathway affects the number of vortices formed in the droplet.^44^ The formation of a single vortex within the droplet, induced by SAWs, influences the movement and aggregation of particles. As the fluid within the droplet rotates due to the vortex, it causes particles suspended in the droplet to move along with it. The vortex motion encourages the particles to move toward the center of the droplet, overcoming forces such as the settling of particles due to gravity and the coffee ring effect, where particles tend to accumulate at the edges of the droplet as it evaporates, as seen in Fig. 4A. This movement towards the center of the droplet due to the vortex causes the particles to aggregate in one spot;^37^ however, multiple vortices, resulting in particles not aggregating in one spot, can be generated when more than half of the droplet is exposed to the SAW. Therefore, to cause a single vortex, a 1 μL droplet of the sample suspension was loaded on the SAW chip such that half of the droplet lay in the SAW propagation pathway, as shown in Fig. 2C.^29^ By maintaining the rotation of the droplet and the aggregation of particles under the proper position of the SAW propagation pathway, the average inter-particle distance decreased due to droplet evaporation, ultimately resulting in the formation of clusters of PSMPs and AuNPs. These clusters are helpful in SERS detection, as aggregated AuNPs on the PSMPs can create hot spots that significantly amplify Raman scattering.

## Results and discussion

### Effects of hydrophobic coating and SAWs on particle aggregation

An analysis of the effect of the hydrophobic coating and SAWs on particle aggregation was performed by observing the distribution of particles, such as PSMPs and AuNPs, left after the droplet evaporated under different conditions.

**Fig. 3:**
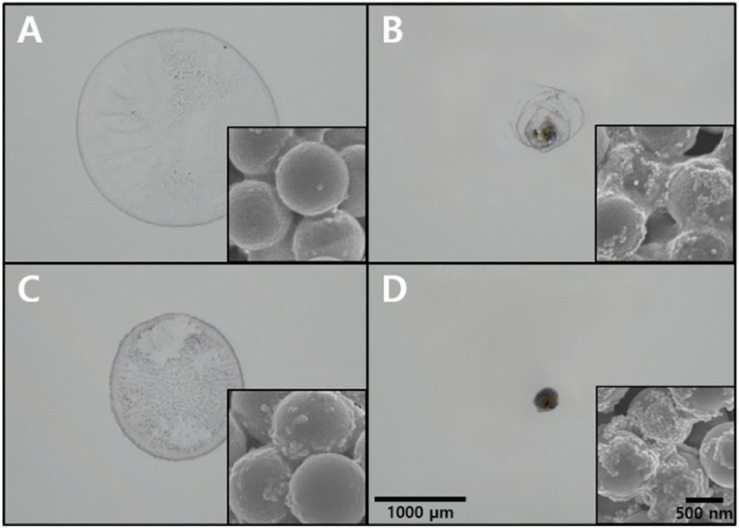
Microscopic and SEM images of aggregated 50 nm AuNPs mixed with 1 μm PSMPs adsorbed with pyrene depending on various conditions: (A) on the original LiNbO_3_ surface without SAWs, (B) on the original LiNbO_3_ surface with SAWs, (C) on the hydrophobic coated LiNbO_3_ surface without SAWs, (D) on the hydrophobic coated LiNbO_3_ surface with SAWs. Each 1 μL of the droplet was loaded on the LiNbO_3_ surface.

Without the hydrophobic coating and SAWs, the particles exhibited a random distribution with no discernible single-point aggregation, and only a few AuNPs adhered to the surface of PSMPs, as shown in Fig. 3A. In this case, there were ∼20 AuNP μm^−2^ on average within an area of ∼2200 μm in diameter. This condition was considered unsuitable for SERS measurements due to the lack of gold nanogaps by particle aggregation. The application of SAWs to this process resulted in a denser distribution of particles, as seen in Fig. 3B. The density of AuNPs within a region of ∼770 μm in diameter was calculated to be ∼220 AuNP μm^−2^. This indicates that SAWs significantly affect the formation of gold nanogaps on the surface of PSMPs by particle aggregation, which could potentially benefit SERS measurements. Fig. 3C shows that the application of a hydrophobic coating alone led to a reduction in the diameter of the aggregated particles compared to the original surface and more AuNPs adhered to the surface of PSMPs than Fig. 3A. AuNPs were distributed at a density of ∼50 AuNP μm^−2^ in an area with a diameter of ∼1480 μm. Nonetheless, the particles were not even densely located in any area. Although it induced a greater density of particles in specific areas, it fell short of producing the single-point aggregation of particles necessary for optimal SERS measurements. The result demonstrated that the hydrophobic coating alone was insufficient for particle aggregation to form gold nanogaps. Finally, the combination of hydrophobic coating and SAWs was studied. This condition created a denser particle distribution, an even smaller diameter of the cluster, and more AuNPs on the surface of PSMPs than in the previous conditions (Fig. 3D). The number of AuNPs was estimated to be ∼1700 AuNP μm^−2^ in a region with a diameter of ∼270 μm. Under this condition, the gap between AuNPs is approximately 1.5 nm, because of a couple of citrate molecules on the interparticle surface.^45^ To achieve the desired formation of gold nanogaps, which are beneficial for SERS measurements, this combined use of hydrophobic coating and SAWs was found to be the most effective approach among all the experimental conditions. The results showed that the particle aggregation varied with the interaction of the surface state of the SAW chips and the internal flow induced by SAWs within the droplet. As the internal flow induced by SAWs rotates not horizontally but with a specific angle (∼22°), called the Rayleigh angle which is caused by the difference in acoustic streaming velocity of the piezoelectric material (LiNbO_3_) and medium (water), the droplet has to maintain a sufficient height to form a vortex.^29,40^ Otherwise, a vortex is not created because the internal flow passes over the top of the droplet without sufficient interaction.^37^ The contact angle of the droplet is relatively higher on the hydrophobic-coated LiNbO_3_ surface (∼105°) than the original LiNbO_3_ surface (∼42°) due to the trichlorosilane monolayer.^46^ As the droplet can maintain its height while evaporating, it contributes to vortex formation. The vortex plays a decisive role in the aggregation of the particles. It keeps the particles moving toward the center of the droplet against the settling of particles and the coffee ring effect.^32^ The application of SAWs and hydrophobic coating appears to be effective for achieving well-aggregated particles into a single point, and is optimal for SERS measurement in this experiment, as the narrow distance between particles creates numerous nanogaps, enhancing Raman scattering.

**Fig. 4:**
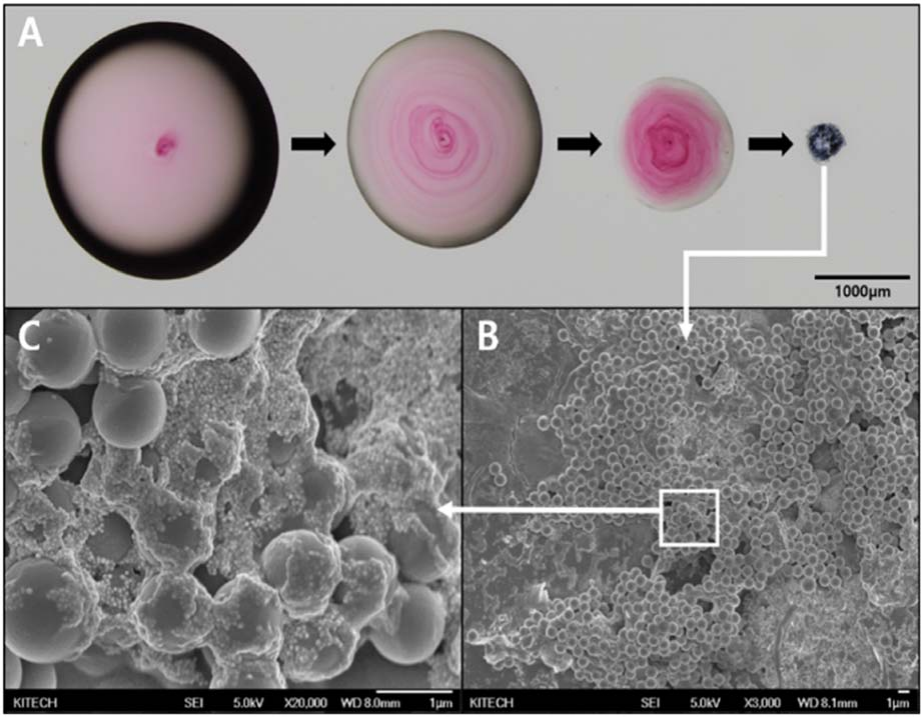
(A) Microscopic images of the aggregation and evaporation process of 1 μL droplet consisting of PSMPs and AuNPs. SEM images of (B) aggregated 1 μm PSMPs and (C) the distribution of 50 nm AuNPs on the surface of the PSMPs.

Fig. 4A shows the evaporation process of the droplet containing PSMPs and AuNPs loaded on the SAW chip. The presence of both SAWs and the hydrophobic coating creates a favorable environment for the particles to aggregate into a single point as the droplet evaporates. Fig. 4B is an SEM image of the cluster that was aggregated after the droplet had evaporated in Fig. 4A. This aggregation is the result of the combined effects of the SAWs and hydrophobic coating, which lead to well-aggregated particles at a single point. Considering the goal of detecting toxic substances adsorbed on microplastic surfaces using SERS, it is crucial to optimize the enhancement of the Raman signal. The formation of gold nanogaps on the PSMP surface, as observed in Fig. 4C, an enlarged image of the PSMP cluster with AuNPs adhering to their surface, plays a critical role in achieving this optimization. The enhancement of Raman signals is predominantly attributed to the localized surface plasmon resonance (LSPR) that occurs when the incident electromagnetic field interacts with metallic nanoparticles, such as AuNPs. LSPR is highly sensitive to the local environment and interparticle distance. When AuNPs are in close proximity, they form nanogaps, leading to a plasmonic coupling between the particles and generating hot spots where the electromagnetic field is considerably amplified. The enhanced electromagnetic field within the hot spots subsequently amplifies the Raman signal of nearby molecules, allowing for their sensitive and selective detection by SERS.^47^ This enhancement is particularly advantageous when detecting trace amounts of toxic substances or differentiating between molecular species. The successful creation of gold nanogaps on the PSMP surface, as shown in Fig. 4C, highlights the effectiveness of the combined approach of SAWs and hydrophobic coating in promoting particle aggregation and nanogap formation. This approach is promising for the detection of toxic substances adsorbed on microplastic surfaces by SERS, allowing the identification and analysis of harmful compounds.

### SERS detection of PAHs

Both PSMPs and PAHs are composed primarily of carbon and hydrogen, and they both have aromatic rings in their structures. This means that they interact strongly with each other, often through the process of adsorption. The adsorption of PAHs on PSMPs can be described by a few key principles. First of all, the primary force that drives the adsorption of PAHs on PSMPs is van der Waals forces, especially London dispersion forces.^48^ These forces are due to the temporary polarization of the electron cloud around a molecule, creating a temporary dipole. This dipole can then induce a similar dipole in an adjacent molecule. Even though each individual interaction is weak, the sum of a large number of these interactions can lead to a significant effect. PSMPs and PAHs are both largely non-polar substances, which means that their electron clouds can easily be temporarily polarized. Additionally, both substances have large, flat aromatic rings, which allow for a large amount of surface contact and therefore strong dispersion forces. This means that PAHs can strongly adsorb on the surface of PSMPs.^49^ Second, molecules naturally move from an area of higher concentration to an area of lower concentration by the second law of thermodynamics. So, when there are more PAH molecules in the solution than on the PSMP surfaces, the PAH molecules will naturally move toward the PSMPs and adsorb on their surface. This adsorption process will continue to do so until there is no longer a difference in concentration between the PAH solution and the PSMP surfaces.^50^

To confirm whether PAHs were adsorbed on PSMPs, sample suspensions consisting of AuNPs and PSMPs adsorbed with pyrene, anthracene, and fluorene were dropped on the SAW chip. Stock solutions of 10 mM pyrene, anthracene, and fluorene were used in this experiment. A 1 μL droplet of the sample suspension was loaded onto the SAW area of the LiNbO_3_ wafer. As the PSMPs and AuNPs in the droplet were mixed and aggregated by the SAWs, gold nanogaps were formed on the surface of the PSMPs. After evaporating the droplet, the SERS spectra were measured at the center of the dried droplet.

**Fig. 5:**
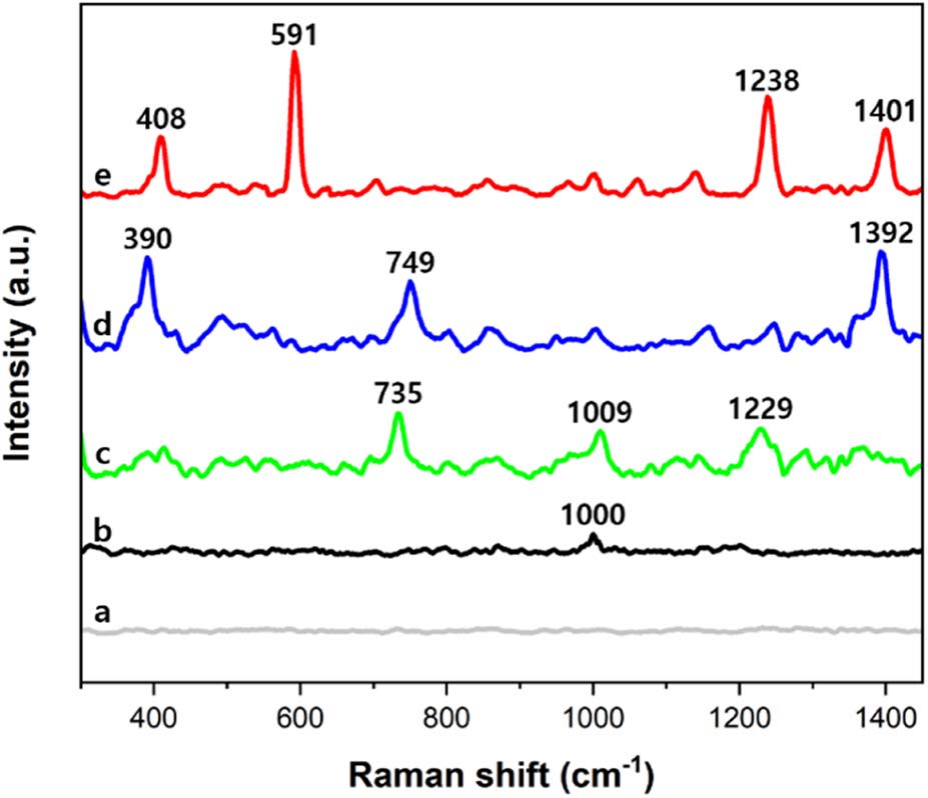
(a) Raman spectrum measured from the cluster of 1 μm PSMPs without AuNPs. SERS spectra measured from the clusters of 50 nm AuNPs and 1 μm PSMPs, which adsorbed (b) no PAHs, (c) fluorene, (d) anthracene, and (e) pyrene. 10 mM PAH solution was used for the PSMPs to adsorb a PAH, respectively. Detected SERS characteristic peaks of each PAH are comparable with their own Raman characteristic peaks.

**Fig. 6:**
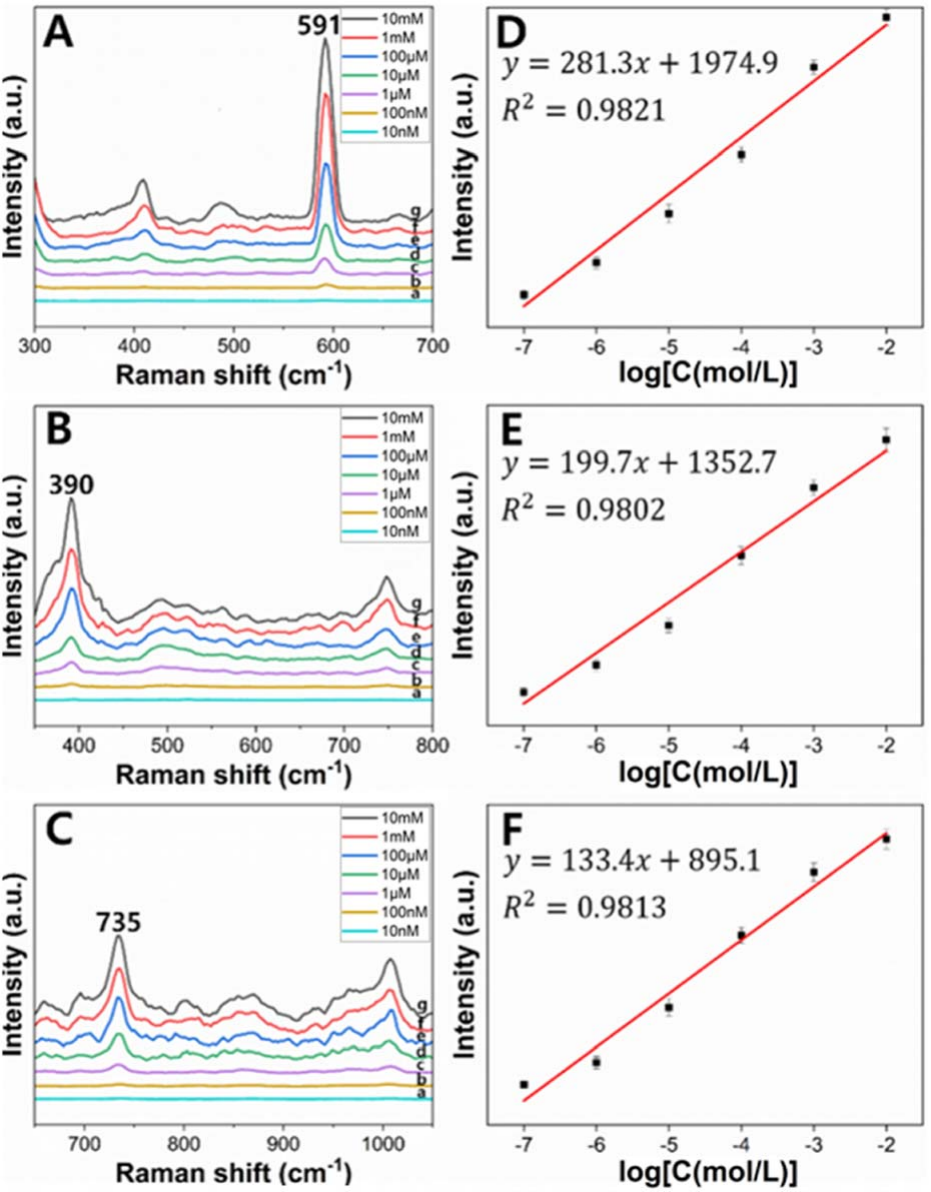
(Left) SERS spectra of three PAHs adsorbed on 1 μm PSMPs at concentrations of (from a to g) 10 nM, 100 nM, 1 μM, 10 μM, 100 μM, 1 mM, and 10 mM (A) pyrene (B) anthracene and (C) fluorene. (Right) Relationship between the SERS intensity of (D) pyrene at 591 cm^−1^, (E) anthracene at 390 cm^−1^, (F) fluorene at 735 cm^−1^ and the logarithm of concentrations of each PAH. Each data point represents the average value from five SERS spectra. Error bars show the sample standard deviations.

Fig. 5a shows that no distinct peak was measured from the cluster of only PSMPs. In SERS spectra measured from clusters of the AuNPs and PSMPs that had not adsorbed any PAHs, no distinct peak was identified except for 1000 cm^−1^, the main characteristic peak of polystyrene, as seen in Fig. 5b.^51^ On the other hand, in SERS spectra measured from clusters of the AuNPs and PSMPs adsorbed with PAHs, characteristic peaks of fluorene, anthracene, and pyrene were identified in the range of 300–1450 cm^−1^, as shown in Fig. 5c–e. As the three PAHs are composed of benzene rings, peak assignments contributing to distinct peaks of the recorded SERS spectra can be determined.^52^ The SERS peaks of the anthracene sample at 1392 cm^−1^ and pyrene sample at 591 cm^−1^ and 1401 cm^−1^ were attributed to ring stretching and breathing. The SERS peaks of the fluorene sample at 1009 cm^−1^ and 1229 cm^−1^ and the pyrene sample at 1239 cm^−1^ were attributed to C–H in-plane bending. The SERS peaks of the fluorene sample at 735 cm^−1^ and the anthracene sample at 749 cm^−1^ were attributed to C–H out-of-plane bending. The SERS peaks of the anthracene sample at 390 cm^−1^ and pyrene sample at 408 cm^−1^ were attributed to C–C and C–H bending. The distinct SERS peaks of the three PAHs were comparable to their Raman characteristic peaks.^12,53–55^ These results indicate that the PAHs had adsorbed on the PSMPs, and their Raman scatterings were enhanced by the gold nanogaps that the SAWs formed on the surface of the PSMPs. Evaluation of the sensitivity of the gold nanogaps formed by the SAWs was also carried out when it comes to SERS detection of the PAHs adsorbed on the PSMPs. It was performed at the range of 10 nM to 10 mM, which is the concentration of the PAH solution used for adsorption on the surface of the PSMPs. Sample suspensions of PSMPs mixed with AuNPs were used. A 1 μL droplet of the sample suspension was dropped onto the SAW chip. After evaporating the droplet, the SERS spectra were measured at the center of the evaporated droplet. Fig. 6 shows the concentration-dependent SERS spectra of pyrene, anthracene, and fluorene at different concentrations. The SERS spectra of pyrene (a)–(g) in the 300–700 cm^−1^ region are shown in Fig. 6A.

The intensity of the peak at 591 cm^−1^ increased with increasing pyrene concentration. The calibration curve of the pyrene peak intensity at 591 cm^−1^ and the pyrene concentration is plotted in Fig. 6D. The pyrene peak intensity varied linearly with the logarithm of pyrene concentration in the range of 10 nM to 10 mM. The calibration curve yielded a reliable linear relationship (*R*^*2*^ = 0.9821). The regression equation of pyrene is *y*_p_ = (281.3 ± 18.9)*x*_p_ + (1974.9 ± 101.9), where *y*_p_ is the SERS peak intensity of pyrene at 591 cm^−1^ and *x*_p_ is the logarithm of the pyrene concentration. The calculated limit of detection (LOD) of pyrene was ∼95 nM. When the same method was used, anthracene and fluorene also showed positive correlations between the SERS intensity and logarithm of the concentration. The regression equation of anthracene and fluorene is *y*_a_ = (199.7 ± 15.6)*x*_a_ + (1352.7 ± 131.1) and *y*_f_ = (133.4 ± 9.2)*x*_f_ + (895.1 ± 44.3), respectively, where the SERS peak intensity of anthracene at 390 cm^−1^ is represented by *y*_a_, and for fluorene at 735 cm^−1^, it is denoted as *y*_f_, and the logarithms of anthracene and fluorene concentrations are expressed as *x*_a_ and *x*_f_. The LOD of anthracene and fluorene was calculated as ∼168 nM and ∼195 nM, respectively (Fig. 6B, C, E and F).

As shown in Fig. 5, it can be seen that the measured SERS spectra show that the SERS intensity of pyrene is greater than that of the other two PAHs, anthracene and fluorene. Furthermore, the calculated LOD of pyrene was smaller than that of the others. This is due to pyrene being composed of four benzene rings compared to anthracene and fluorene, which are composed of two or three benzene rings. As the number of benzene rings increases, the molecule becomes larger and has more electrons. This increases the surface area where PAH molecules come into contact with PSMPs and induces stronger van der Waals forces, especially London dispersion forces, allowing the PAH molecules to be better adsorbed on the PSMP surface. Therefore, pyrene with more benzene rings is better adsorbed on PSMPs at the same concentration than other PAHs, resulting in a greater SERS intensity and smaller LOD.

### Identification of PAHs in the mixture

Since a real sample contains various PAHs, the capability to distinguish individual PAHs in mixtures was tested. To adsorb the PAHs on the PSMPs, three 10 mM stock solutions of pyrene, anthracene, and fluorene were mixed in the same volume so that the total concentration of the solution was 10 mM. A suspension of PSMPs adsorbed with three PAHs and AuNPs was used in this process. A 1 μL droplet of the suspension was loaded onto the SAW chip, and the SERS spectrum of the mixture of three PAHs was collected after evaporating the droplet.

**Fig. 7:**
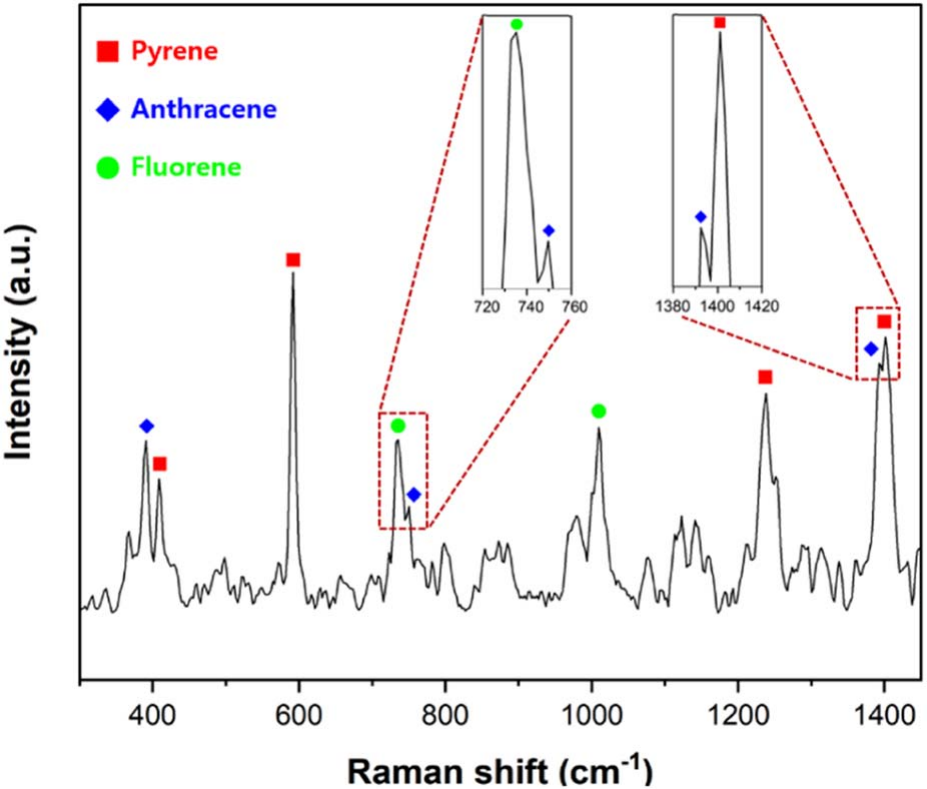
SERS spectrum of a mixture of 3 PAHs (pyrene, anthracene, fluorene) with a total concentration of 10 mM adsorbed on 1 μm PSMPs. Discriminant peaks of each PAH are labeled with different symbols. Peaks with similar shifts are partially overlapped and observed as broad peaks.

As shown in Fig. 7, the discriminant peaks of each PAH are labeled with different symbols. The characteristic peaks of each PAH are labeled with different symbols. The characteristic Raman peaks of anthracene at 390, 749, and 1392 cm^−1^ appeared on the SERS spectrum. The SERS peaks of fluorene at 735 and 1009 cm^−1^ were consistent with the Raman peaks of fluorene. The SERS peaks of pyrene at 408, 591, 1238, and 1401 cm^−1^ matched well with those of pyrene.^12,53–55^ Although pyrene, anthracene, and fluorene contain fused benzene rings and have similar chemical and physical properties, the spectrum shows that all three PAHs adsorbed on the PSMPs could be discriminated successfully. As the system demonstrated the ability to detect and distinguish toxic substances adsorbed on microplastics, it can be applied to analyze a mixture of surface-adsorbent toxic substances.

## Conclusion

In this study, we introduce a system for detecting toxic substances adsorbed on microplastic surfaces using SERS and SAWs. The application of SAWs and hydrophobic coating enabled the effective aggregation of particles, forming gold nanogaps that enhanced the Raman signal. The system was tested on three PAHs, pyrene, anthracene, and fluorene, adsorbed on PSMPs and demonstrated the ability to detect and distinguish these PAHs. The SERS intensity and logarithm of the concentration of PAHs showed a positive correlation in the range of 10 nM to 10 mM with good linearity (*R*^*2*^ = 0.98). The limits of detection for pyrene, anthracene, and fluorene were determined to be around 95, 168, and 195 nM, respectively. Unlike existing detection methods, since the extraction process, which can be time-consuming and needs solvents, is not required, our proposed system allows for rapid detection of which toxic substances are adsorbed on microplastics and does not produce additional contamination. This study has implications for environmental monitoring as it can be utilized to detect and distinguish toxic substances adsorbed on microplastics in various ecosystems. The developed system is expected to be applicable to water quality assessments, pollutant monitoring, and tracking the spread of surface-adsorbent toxic substances in aquatic environments.

## Conflicts of interest

There are no conflicts to declare.

## Supplementary Material
